# Predicting coping with expectation violations: combining the ViolEx Model and the Covariation Principle

**DOI:** 10.3389/fpsyg.2023.1152261

**Published:** 2023-05-23

**Authors:** Martin Christian Pietzsch, Martin Pinquart

**Affiliations:** Department of Psychology, Philipps University of Marburg, Marburg, Germany

**Keywords:** ViolEx Model, expectation violation, Covariation Principle, vignette study, experiment, assimilation, accommodation, immunization

## Abstract

An experimental vignette study examined whether three specific situational cues predict ways of coping with violated expectations. The situational cues (consistency, distinctiveness, consensus) were derived from the Covariation Principle. The assessed coping strategies were based on the ViolEx Model—assimilation (activities to fulfill one’s expectation), accommodation (expectation change) and immunization (ignoring the discrepant information). A sample of 124 adults (mean age = 23.60 years; 49.19 percent psychology students) were randomly assigned to an experimental and control condition. Participants of the experimental condition read several vignettes about expectation violations with systematically manipulated situational cues, while participants of the control condition received the same vignettes without such cues. Participants had to rate the usefulness of each coping strategy per vignette. The situational cues mostly led to response shifts in coping tendencies: Situations with low consistency cues mostly led to immunization, whereas high consistency led to assimilation in the case of high distinctiveness and to accommodation in the case of low distinctiveness. Consensus cues only played a minor part in the coping process. The results show that situational characteristics influence people’s coping behavior, regardless of their dispositional preferences for certain coping strategies.

## 1. Introduction

Expectation violations are a common everyday phenomenon and each day individuals have to cope with situations, in which their expectations do not come true. The ViolEx Model (Rief et al., 2015; Gollwitzer et al., 2018) proposes three ways of coping with expectation violations: Assimilation (activities aimed at fulfilling one’s expectation in the future), accommodation (i.e., adapting the former expectations to the current unexpected outcome), and immunization (devaluing or reframing the meaning of the current expectation violation) (Gollwitzer et al., 2018, p. 2). The use of these coping processes is likely to depend upon situational characteristics as well as personality dispositions (Pinquart et al., 2021). When confronted with expectation violations, individuals might look for certain characteristics that coincide with such events—in order to understand the cause of an expectation violation, as this information is relevant for predicting whether similar expectation violations may occur in the future and/or whether such events could be prevented. If expectation violations refer to events that may happen repeatedly to oneself and other people (e.g., in the case of achievement tests at school or university), one might use the Covariation Principle and related situational cues—originally introduced by Kelley (1967, 1973)—for predicting and finding a suitable way of coping with such expectation violations. While Kelley and Michela (1980) already mention possible links between expectations and attributions, the present study is the first to connect the ViolEx Model with the Covariation Principle.

The Covariation Principle proposes that three situational factors are relevant for drawing conclusions about causality: consensus (i.e., whether similar things happened to many other people), distinctiveness (i.e., whether things happened only in a specific situation), and consistency (i.e., whether things happened repeatedly in the same way; Kelley, 1973). In the combination of low consensus, low distinctiveness, and high consistency (things happen repeatedly and across many situations only to an individual person), these outcomes will be attributed to characteristics of the person (internal attribution). In the combination of high consensus, high distinctiveness, and high consistency, the outcomes will be attributed to characteristics of the situation (external attribution). In contrast, low consistency within a situation will promote an attribution to (exceptional) circumstances, regardless of whether consensus and/or distinctiveness are high or low (McArthur, 1972; Kelley, 1973). In case of an attribution to (exceptional) circumstances it seems to be very unlikely that the particular outcome might occur again in future situations, as it is inconsistent with former experiences. Apart from these, Kelley (1973) did not make assumptions about every possible combination of the three cues. Therefore, we will add our own assumptions about the remaining two combinations and relate them to certain coping processes from the ViolEx Model.

Applying the Covariation Principle to coping with expectation violations, immunization will be most likely happening in the case of low consistency (as the expectation has often been confirmed in the past, but not in the present, expectation-violating case). Thus, the present expectation violation is easily perceived as an “exception from the rule,” and one does not need to change his or her expectation because the typical (expectation-confirming) situations are likely to occur in the future. In fact, Filipowicz et al. (2018) found that a single expectation violation after a series of expectation confirmations was perceived as an exception, and many persons were not willing to change their expectation unless they experienced a series of expectation violations. Thus, expectation change (accommodation) should be most likely to occur if the expectation has already been disconfirmed several times in the past (high consistency), and across different situations (low distinctiveness, lack of triggers for defining the specific situation as an exception). Under these circumstances, maintaining one’s expectation would very likely provoke more expectation violations in the future. While for this combination of high consistency and low distinctiveness, Kelley (1973) only made assumptions about situations with low consensus, we expand our assumptions upon expectation violations with high consensus (i.e., if these situations also happen to other people), as the perception of other people receiving that same expectation-violating outcome consistently across many different situations might also lead to accommodation, i.e., expectation change.

In the case of consistent expectation disconfirmation, future expectation violations can not only be avoided by accommodating one’s expectation but also by actively seeking future expectation confirmations (assimilation). However, assimilative behavior presupposes that the individual has some control over the occurrence of future expectation confirmations (Pinquart et al., 2021).

If the expectation violation happens only in a distinctive situation which is different from expectation-confirming settings, an individual might try to change aspects of that specific type of situation (with the intention to promote future expectation-confirming outcomes). Furthermore, if the expectation violation is limited to a specific (i.e., distinctive) situation, it might be easier to find a suitable assimilative coping strategy than for broader contexts. Thus, we assume that assimilative behavior will be promoted by the combination of high consistency and high distinctiveness. While with regard to this particular combination of cues, the original Covariation Principle by Kelley (1973) only made assumptions about attributions in situations with high consensus, we also expect increased assimilative behavior after expectation violations in situations with low consensus, as expectation violations that repeatedly happen to only a single person (or limited number of people) may allow a very specific assimilative coping behavior (if one has control over the outcome).

In our study, we use two ways of empirically testing associations between situational cues and ways of coping with expectation violations. First, we test whether a particular coping strategy is preferred over the other strategies within a particular combination of situational cues (comparison between strategies; hypotheses 1a, 2a, and 3a). Second, we test whether a particular coping strategy is more often preferred in the case of a specific combination of situational cues than in other combinations of these cues (comparison between situations; hypotheses 1b, 2b, and 3b). Kelley and Michela (1980) conceptually compared the covariation principle to an ANOVA, as there might be specific main and/or interaction effects of the cues. Therefore, our study examines the following hypotheses:

*1a*: Immunization is preferred over the other two strategies in expectation-violating scenarios with low consistency (regardless of whether distinctiveness and/or consensus are high or low).

*1b*: Immunization ratings are generally higher in scenarios with low consistency than in scenarios with high consistency (regardless of whether distinctiveness and/or consensus are high or low).

*2a*: Accommodation is preferred over the other two strategies if expectation-violating scenarios are characterized by the combination of high consistency and low distinctiveness (regardless of whether consensus is high or low).

*2b*: Accommodation ratings are generally higher in scenarios characterized by the combination of high consistency and low distinctiveness than in scenarios with any other combination of these cues (regardless of whether consensus is high or low).

*3a*: Assimilation is preferred over the other strategies if expectation-violating scenarios are characterized by the combination of high consistency and high distinctiveness (regardless of whether consensus is high or low).

*3b*: Assimilation ratings are generally higher in situations characterized by the combination of high consistency and high distinctiveness than in scenarios with any other combination of these cues (regardless of whether consensus is high or low).

Hence, the preference for the expected coping strategy within a given cue combination should not only be higher than the other coping strategies within that cue combination, but also higher than the usage of that same strategy within other cue combinations. As we specified our hypotheses without expecting any influence of consensus, one could argue to leave this cue out. However, we decided to also include this third cue as we did not want to miss any unexpected results. Thus, our study included all three cues of the Covariation Principle (Kelley, 1973). An overview of all cue combinations in relation to the respective hypotheses can be found in Table 1.

**Table 1:** Overview of all cue combinations and coping strategies in relation to the hypotheses.

	Situational cues			Expected preferred coping strategy
	Consensus	Distinctiveness	Consistency	
Hypothesis 1	High	High	Low	Immunization
	High	Low	Low	Immunization
	Low	High	Low	Immunization
	Low	Low	Low	Immunization
Hypothesis 2	Low	Low	High	Accommodation
	High	Low	High	Accommodation
Hypothesis 3	High	High	High	Assimilation
	Low	High	High	Assimilation

Combination of situational cues from the Covariation Principle and related ViolEx coping strategies are presented in the order of the underlying hypotheses. This does not resemble the order in which the cue combinations were actually presented in our study.

## 2. Materials and methods

The hypotheses stated above were tested with a vignette study—as this study design was also used by McArthur (1972) for investigating the role of the situational cues mentioned above. Before the main study, we conducted a pre-study (designed within a student course) to test the practicality of the study design. The main study was created upon the insights gained from the pre-study and covered a larger number of vignettes. The timespan between the two studies was longer than a year, therefore we did not expect any remembrance effects if any participants might have taken part in both studies. Both studies were conducted in German wording, all quotations given below are translated from the original content. Before the beginning of the study, participants received written information about the procedure and were asked to provide their consent to these terms. Participants who did not provide their consent were not able to take part in the study. The study was approved by the local ethics committee in advance.

At the beginning of the study, participants were randomly assigned to either an experimental or control group. Participants of the experimental group received 16 short stories containing manipulated cues about the distinctiveness, consensus and consistency (high vs. low) of a certain expectation-violating event (2 × 2 × 2 design). Each cue combination was presented twice. However, vignettes with the same combination of cues were never presented directly one after another but in an initially randomized order. This procedure also reduced the risk that certain results might mainly be related to the specific backstory of a single vignette, rather than to the combination of cues themselves. While Kelley’s original study focused on attributions, the new experiment used vignettes to describe situations where a person’s expectations are violated. The study only included everyday stories with worse than expected events, as the valence of expectation violation would otherwise be a confounding factor (see Pinquart et al., 2021). Three exemplary vignettes in German wording and English translations with additional explanations of the respective included cue combinations can be found in Supplementary material S1, all original vignettes are publicly available (see data availability statement). All stories were written in a third person perspective, and participants had to rate on 4 point Likert scales, how the main character of each story should cope with the situation: Below each vignette, we presented the general question “How should the person cope with the violated expectation?” and the participants had to rate the usefulness of each of the three ViolEx coping strategies from 1 (“not useful”) to 4 (“very useful”). The strategies were “Do something, so that the own expectations will come true in the future” (referring to assimilation), “Adapt the own expectations to the current situation” (referring to accommodation) and “Treat the current situation as an exception from the rule” (referring to immunization). Though immunization can be conceptualized in two different forms (data-oriented and concept-oriented immunization; Gollwitzer et al., 2018), it was decided to use only one item with a focus on data-oriented immunization, as this was more suitable to the current study and did not lead to an overrepresentation of immunization items.

Immediately after rating the usefulness of the three coping strategies on Likert scales—i.e., directly below the rating items and using the same strategy wordings as above—the participants had to rank the three strategies “beginning with the one you consider most useful in the current situation.” This was used mainly to have specific information on participants’ preferred coping strategy for each situation, in addition to the ratings stated above. The two different types of items were analyzed separately.

Additionally, further items were presented in which participants were asked to rate certain characteristics of the vignette as a manipulation check: These items included the situational cues (consensus, distinctiveness, consistency; in easy-to-understand phrasing) with rating scales from 1 (“Not true”) to 4 (“Completely true”). Each vignette and the respective items were presented together, but only one vignette per page. For each story, the same set of items was presented. Therefore, the wording of the items did not vary between vignettes. At the end of the study, participants were also asked to give some demographical information and to rate whether they were able to understand the content of the vignettes.

The control group received the same 16 vignettes as the experimental group, but without the relevant cues described above. Instead, each vignette was expanded with irrelevant information, so that there was no difference in vignette length or reading time between experimental and control group. We did not have any specific hypotheses about coping preferences within the control group, as the effects of the situational cues within the experimental group were the focus of our study. The purpose of the control group was to test whether the expected coping ratings were higher in the experimental group than in the control group, just to make sure any statistical effects could be attributed to the presence of cues within the experimental group. Therefore, participants within the control group also received the same general items as participants within the experimental group.

As Pinquart et al. (2021) already assumed, there might also be individual dispositional tendencies to prefer a certain coping strategy (i.e., immunization, assimilation, accommodation) over the others. Such dispositions might influence the situational coping process, especially if there are no situational cues that promote a particular way of coping, but also to a lesser degree in a situation where such cues are given. We therefore controlled for these dispositions with regard to overall rating tendencies across all vignettes, as will be described in the Results section.

The study was conducted from December 2021 until January 2022, in an online format via the German platform SoSci Survey (Leiner, 2022). Participation took about 60 min. The recruitment procedure included the official university student mailing list, but also a call on social media and some other mailing lists. Participants could either register to gain course credits or take part in a raffle to win one of four 25€ vouchers. We included all participants who responded to the items of all 16 vignettes. Therefore, 28 participants were not included in the sample, as they dropped out from the study, mostly after the first few items. Out of the remaining 129 participants, further five people were excluded, as they stated problems understanding the content of the vignettes (three people from the experimental group, two from the control group, each with a self-rating of 1 or 2 on the four-point text comprehension item). The final analysis sample consisted of *N* = 124 participants (94 females, 30 males). The experimental group (*n* = 62) and the control group (*n* = 62) did not differ significantly in age, gender and reported text comprehension. The mean age was 23.60 years (*SD* = 7.07); 49.19 percent of the participants were psychology students.

For data analysis we used SPSS Version 27 (IBM, 2020), the data file and analysis code are publicly available (see data availability statement). As we occasionally used multiple tests per hypothesis in order to analyze all details of our data, we decided to decrease our critical alpha level below the commonly used value of *α* = 0.05. In order to keep our analysis coherent, we generally used *α* = 0.01 as critical cut-off for *p*-values. Additionally, within the ANOVAs and other multi-variable-tests, the alpha levels were mostly adjusted by the software, using the Bonferroni method (IBM, 2020), e.g., to correct the post-hoc tests.

## 3. Results

Before our main analyses, we evaluated the cue ratings from the manipulation check. In order to test whether high (or low) cues had actually been perceived as intended within the experimental group, we compared the mean perceptions of consensus, distinctiveness, and consistency between vignettes representing high versus low scores of these cues. Three *t*-tests—one per cue—revealed that vignettes with high cues were actually rated significantly higher on this dimension than vignettes with low cues [consensus: *t*(61) = 10.65, *p* < 0.001, *d* = 1.35; distinctiveness: *t*(61) = 8.61, *p* < 0.001, *d* = 1.09; consistency: *t*(61) = 18.75, *p* < 0.001, *d* = 2.38]. Thus, the vignettes were perceived as intended by the experimental group. The mean cue ratings from the manipulation check can be found in Supplementary material S2.

As a first step of testing hypotheses 1a, 2a, and 3a, we analyzed the coping strategy choices by use of the ranking items. In order to compare the ordinal-scaled preferences within each experimental group vignette, we calculated Friedman rank tests. For 13 of 16 vignettes most of the participants preferred the expected coping strategy, of which for five vignettes this preference was significantly higher than both other strategies and for eight vignettes significantly higher than only the lowest ranked strategy. For three vignettes the preferred choice was different from our hypotheses, in each of these the participants preferred assimilation instead of the expected strategy.

For comparisons of coping strategy choices between the experimental condition and the control condition, we used *U*-tests. Within the 13 vignettes that matched our expected coping strategies, responses on eight vignettes showed a significantly higher preference for that strategy in the experimental condition compared to the control group. Within each of the three vignettes that deviated from our presumptions, there were no significant differences for the expected coping strategy between experimental and control group. The detailed results of the ranking item analyses can be found in Table 2.

**Table 2:** Analysis of the most preferred strategy choice per vignette.

Vignette	Cues^a^	Expected coping strategy	Frequencies of strategy use (experimental group)			Control group comparison^b^
			Ass.	Acc.	Imm.	
A	+ + −	Immunization	25	4^d^	*33^d^*	10**
B	− + +	Assimilation	*42^d^*	14	6^d^	12**
C	− − +	Accommodation	22	*40^d^*	0^d^	31
D	− + −	Immunization	4^c^	2^d^	*56^cd^*	42*
E	+ + +	Assimilation	*30^d^*	24	8^d^	17*
F	+ − −	Immunization	31^d^	8^d^	*23*	23
G	+ − +	Accommodation	15^c^	*46^cd^*	1^d^	23**
H	− − −	Immunization	40^d^	6^d^	*16*	11
I	+ − −	Immunization	4^d^	21	*37^d^*	14**
J	− + +	Assimilation	*49^cd^*	8^c^	5^d^	46
K	− + −	Immunization	5^d^	7^c^	*50^cd^*	15**
L	+ − +	Accommodation	26	*31^d^*	5^d^	23
M	+ + +	Assimilation	*51^cd^*	5^d^	6^c^	52
N	+ + −	Immunization	3^d^	24	*35^d^*	15**
O	− − +	Accommodation	33^d^	*26*	3^d^	19
P	− − −	Immunization	4^d^	28	*30^d^*	33

Frequencies of expected coping strategies within the experimental group are formatted in italics.

^a^Pre-specified combination of Consensus, Distinctiveness, and Consistency, respectively, each high or low. ^b^Frequency of the hypothesized coping strategy in the control group. The asterisks indicate whether the frequency of use of this strategy differs significantly between experimental and control group (*U*-test). ^c^Significant difference between most frequently and second most frequently chosen way of coping at *p* < 0.01 (Friedman-test). ^d^Significant difference between most frequently and least frequently chosen way of coping at *p* < 0.01 (Friedman-test).

*U-test significant at *p* < 0.01, **U-test significant at *p* < 0.001.

When comparing coping strategies within the experimental group, the effects of the situational cues and of dispositional preferences for coping strategies (that are independent of such cues) overlap. However, as we only wanted to analyze the situational differences—depending on the individual vignettes and respective cues—any unwanted dispositional influences should be removed from our calculations. Therefore we continued our analysis with the rating items, in order to control for such dispositional tendencies (as will be described below). For a first overview we compared the three mean strategy ratings within the experimental group in a one-way ANOVA. The highest mean was found for assimilation, with significant preference for assimilation over immunization and for accommodation over immunization, but only a descriptive preference for assimilation over accommodation [*F*(2, 122) = 25.16, *p* < 0.001, η*_p_*^2^ = 0.29]. To control for the dispositional tendencies, we centered each coping strategy on a per-participant-level: We calculated each participant’s rating mean (across all vignettes) per strategy and subtracted this value from each single vignette rating, leaving only the deviations from their individual means. The rating values noted below therefore display the participants’ situational response shifts from their typical (i.e., dispositional) rating behavior: Values above zero indicate a higher-than-average rating, whereas values below zero indicate a lower-than-average rating. The scaling distances, however, remain the same as for the original scale (e.g., a value of +1 equals a preference of one scale point above the individual strategy mean).

As a first analysis of general response shifts across all experimental group vignettes, we assessed the main and interaction effects of the included situational cues. Therefore we calculated mean values from the respective four vignettes with the same combination of distinctiveness and consistency cues (high vs. low; additional effects of consensus cues were analyzed at a later point, see below), resulting in four different means per strategy, independently for experimental and control group. In order to test the hypotheses 1b, 2b, and 3b, we calculated three two-factor repeated-measures ANOVAs with distinctiveness and consistency (each high vs. low) as independent variables, one ANOVA per coping strategy, within the experimental group. For assimilation, we found a significant main effect of consistency [*F*(1, 61) = 226.60, *p* < 0.001, η*_p_*^2^ = 0.79], i.e., assimilation was higher for vignettes with high (*m* = 0.41) than with low consistency (*m* = −0.41), and a significant interaction effect of distinctiveness and consistency [*F*(1, 61) = 25.16, *p* < 0.001, η*_p_*^2^ = 0.29], with the combination of high distinctiveness and high consistency showing the highest assimilation scores (*m* = 0.57). For accommodation, we found significant main effects of both distinctiveness [*F*(1, 61) = 86.27, *p* < 0.001, η*_p_*^2^ = 0.59], i.e., accommodation was higher for vignettes with low (*m* = 0.26) than with high distinctiveness (*m* = −0.26), and consistency [*F*(1, 61) = 26.60, *p* < 0.001, η*_p_*^2^ = 0.30], i.e., accommodation was higher for vignettes with high (*m* = 0.22) than with low consistency (*m* = −0.22), and a significant interaction effect of distinctiveness and consistency [*F*(1, 61) = 24.99, *p* < 0.001, η*_p_*^2^ = 0.29], with the combination of high consistency and low distinctiveness being associated with the highest accommodation scores (*m* = 0.61). For immunization, we found significant main effects of both distinctiveness [*F*(1, 61) = 77.66, *p* < 0.001, η*_p_*^2^ = 0.56], i.e., immunization was higher for vignettes with high (*m* = 0.21) than with low distinctiveness (*m* = −0.21), and consistency [*F*(1, 61) = 352.11, *p* < 0.001, η*_p_*^2^ = 0.85], i.e., immunization was higher for vignettes with low (*m* = 0.80) than with high consistency (*m* = −0.80), and a significant interaction effect of distinctiveness and consistency [*F*(1, 61) = 9.21, *p* = 0.004, η*_p_*^2^ = 0.13], with the highest immunization scores for the combination of low consistency and high distinctiveness (*m* = 1.08). These results generally support our hypotheses. However, the two-factor ANOVA effects do not only include the actually preferred coping strategies (i.e., the highest ratings for each cue combination), but might also be driven by response shifts within the less preferred coping strategies. From a practical perspective, one can assume that mainly the most-preferred coping strategy would be behaviorally relevant, while the less preferred would be less likely to be observed, especially in the case of any significant differences between strategy preferences. Therefore, we continued our analysis with a focus on the direct comparison of ratings within and between the respective cue combinations.

**Figure 1:**
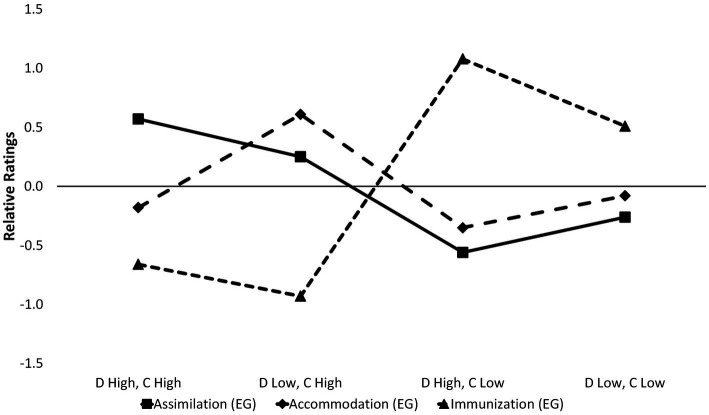
Strategy ratings depending on cue combinations of distinctiveness and consistency. The figure shows the relative ratings for each coping strategy within the experimental group for the combinations of (high vs. low) distinctiveness (labeled “D”) and (high vs. low) consistency (Labeled “C”). The y-axis shows the deviation from each mean rating, a value of zero represents the mean rating across all four cue combinations.

In order to test for significant differences between the cue combination means described above, we calculated additional one-way repeated measures ANOVAs with additional post-hoc tests between the combinations for each coping strategy within the experimental group. The analyses revealed that for assimilation the combination “high consistency, high distinctiveness” (*m* = 0.57) showed significantly higher ratings than any other cue combination [*F*(3, 183) = 87.00, *p* < 0.001, η*_p_*^2^ = 0.59]. For accommodation, the combination “high consistency, low distinctiveness” (*m* = 0.61) showed significantly higher ratings than any other cue combination [*F*(3, 183) = 40.98, *p* < 0.001, η*_p_*^2^ = 0.40]. For immunization, both cue combinations with “low consistency” showed significantly higher ratings than combinations with “high consistency” [*F*(3, 183) = 227.14, *p* < 0.001, η*_p_*^2^ = 0.79], but the combination “low consistency, high distinctiveness” (*m* = 1.08) was also significantly higher than “low consistency, low distinctiveness” (*m* = 0.51). An overview can be found in Figure 1.

As a last step within the general analysis of effects of distinctiveness and consistency, we tested within each cue combination whether the expected coping strategy ratings were significantly higher than ratings of the other coping strategies, as well as the respective control group ratings for the expected preferred coping strategies. The results showed that each expected coping strategy was significantly higher than the other possible coping strategies, and also significantly higher than its respective control group variant, with all relevant *p*-values below *p* < 0.001 and effect sizes that can be interpreted as “large” (η*_p_*^2^ > 0.14; *d* > 0.80) according to the taxonomy by Cohen (1988). An overview of these results can be found in Table 3.

**Table 3:** Analysis of ratings within two-factor cue combinations (distinctiveness and consistency).

Cues^a^	Expected coping strategy	Ratings experimental group (One-way ANOVA)				Control group (CG) comparison^b^	
		Ass.	Acc.	Imm.	η*_p_*^2^	Rating of expected strategy in CG	*d*
+ +	Assimilation	*0.57^cd^*	−0.18^c^	−0.66^d^	0.68	0.22**	0.88
− +	Accommodation	0.25^c^	*0.61^cd^*	−0.93^d^	0.74	0.26**	0.84
+ −	Immunization	−0.56^d^	−0.35^c^	*1.08^cd^*	0.74	−0.06**	2.54
− −	Immunization	−0.26^d^	−0.08^c^	*0.51^cd^*	.51	0.16**	0.92

The means of expected coping strategies within the experimental group are formatted in italics.

^a^Pre-specified combination of distinctiveness and consistency, respectively, each high or low. ^b^Rating of the hypothesized coping strategy in the control group. The asterisks indicate whether the this rating differs significantly between experimental and control group (independent samples *t*-test, one-tailed). ^c^Significant difference between highest and second highest rating at *p* < 0.01. ^d^Significant difference between highest and lowest rating at *p* < 0.01. η*_p_*^2^ effect size for the comparison of ratings within the experimental group (one-way ANOVA). *d* effect size for the comparison of the expected coping strategy rating between experimental group and control group (independent samples *t*-test, one-tailed).

**t-test significant at *p* < 0.001.

On a single-vignette level, the analysis of the situational response shifts within the experimental group showed that for 15 of the 16 vignettes, the ratings significantly matched our hypotheses: For eight vignettes, the expected strategy was significantly higher than both other strategies, while for seven vignettes, the expected strategy was significantly higher than only the lowest rated strategy. One vignette, however, showed a significant preference for assimilation instead of the expected preference for immunization (see Table 4, vignette H).

**Table 4:** Analysis of rating items within single vignettes.

Vignette	Cues^a^	Expected coping strategy	Ratings experimental group (One-way ANOVA)				Control group (CG) comparison^b^	
			Ass.	Acc.	Imm.	η*_p_*^2^	Rating of expected strategy in CG	*d*
A	+ + −	Immunization	0.34	−0.49^d^	*0.83^d^*	0.39	−0.51**	1.60
B	− + +	Assimilation	*0.49^cd^*	−0.46^c^	−0.65^d^	0.25	−0.48**	0.94
C	− − +	Accommodation	0.33	*0.67^d^*	−0.91^d^	0.55	0.38	0.36
D	− + −	Immunization	−0.61^c^	−0.62^d^	*1.41^cd^*	0.67	0.77**	1.02
E	+ + +	Assimilation	*0.15^d^*	−0.02	−.51^d^	0.13	−0.29*	0.49
F	+ − −	Immunization	0.26	−0.20^d^	*0.60^d^*	0.15	0.09*	0.58
G	+ − +	Accommodation	−0.27^c^	*0.59^cd^*	−1.10^d^	0.48	0.31	0.32
H	− − −	Immunization	0.66^d^	−0.63^d^	*0.19*	0.36	0.17	0.02
I	+ − −	Immunization	−1.03^d^	0.17^c^	*0.77^cd^*	0.51	0.02**	0.81
J	− + +	Assimilation	*0.81^cd^*	0.21^c^	−0.78^d^	0.54	0.80	0.01
K	− + −	Immunization	−0.66^d^	−0.36^c^	*1.22^cd^*	0.60	−0.33**	1.91
L	+ − +	Accommodation	0.42	*0.61^d^*	−0.83^d^	0.56	0.15**	0.66
M	+ + +	Assimilation	*0.84^cd^*	−0.44^c^	−0.70^d^	0.52	0.87	−0.05
N	+ + −	Immunization	−1.32^d^	0.06^c^	*0.88^cd^*	0.58	−0.14**	1.14
O	− − +	Accommodation	0.52	*0.58^d^*	−0.88^d^	0.55	0.20*	0.53
P	− − −	Immunization	−0.93^d^	0.32	*0.48^d^*	0.43	0.35	0.14

The means of expected coping strategies within the experimental group are formatted in italics.

^a^Pre-specified combination of Consensus, Distinctiveness, and Consistency, respectively, each high or low. ^b^Rating of the hypothesized coping strategy in the control group. The asterisks indicate whether the this rating differs significantly between experimental and control group (independent samples *t*-test, one-tailed). ^c^Significant difference between highest and second highest rating at *p* < 0.01. ^d^Significant difference between highest and lowest rating at *p* < 0.01. η*_p_*^2^ effect size for the comparison of ratings within the experimental group (one-way ANOVA). *d* effect size for the comparison of the expected coping strategy rating between experimental group and control group (independent samples *t*-test, one-tailed).

*t-test significant at *p* < 0.01, **t-test significant at *p* < 0.001.

Additionally, we compared the ratings for the expected coping strategies between experimental and control group per vignette. Within the 15 vignettes that matched our presumptions, 10 vignettes showed significantly higher ratings for the experimental group, whereas there were no significant group differences for the other remaining vignettes. For the one vignette that did not match our hypothesis (vignette H, see above), there was also no significant difference between experimental and control group. The detailed rating results per vignette can be found in Table 4.

As a final step of our analysis, we tested whether there were any effects of consensus cues by analyzing coping strategy rating means from the respective two vignettes with the same combination of consensus, distinctiveness and consistency cues, in a similar way as we analyzed the two-cue means (see above). The extended results of this exploratory analysis can be found in Supplementary material S3. Our main focus was to find out whether there were differences for the preferred coping strategies between the high and low consensus variants within the respective cue combinations of distinctiveness and consistency. As for the two-cue analysis, we calculated one-way repeated measures ANOVAs between the three-cue combinations, individually for each coping strategy within the experimental group. Post-hoc analyses showed that the cue combination “distinctiveness high, consistency high” was associated with the highest assimilation ratings, with no significant difference between the high and low consensus variants. For the significantly highest accommodation ratings, which we found for “distinctiveness low, consistency high,” there was also no significant difference between the high and low consensus variants. Within immunization ratings with low consistency cue combinations, there were some significant differences, but all of the immunization ratings within low consistency cue combinations were significantly higher than those within high consistency cue combinations. The highest immunization ratings were found for “low consensus, high distinctiveness, low consistency.” Therefore, these exploratory findings about consensus are not contradictory to our presumptions.

## 4. Discussion

### 4.1. General discussion

Coping with expectation violations is a process that is influenced by both situational and dispositional factors (Pinquart et al., 2021). As our results suggest, certain combinations of situational cues, derived from the Covariation Principle, predicted the use of specific coping strategies. Our study showed that certain combinations of these cues can be used to promote specific coping strategies (in support for hypotheses 1a, 2a, 3a), and that especially the consistency between the current situation and former experiences, as well as the distinctiveness of the situation are relevant for the choice of the coping strategy (in support for hypotheses 1b, 2b, 3b). However, this has to be viewed as a relative response shift rather than an absolute preference. Additionally, there are stable individual tendencies that may generally lead to people preferring one coping strategy over the other.

Our results regarding situational factors can be summarized as follows: If the expectation violation happens repeatedly over time (high consistency) and only in a particular context (high distinctiveness), there is an increased tendency to become active in order to prevent future expectation violations (assimilation). If the expectation violation happens repeatedly over time (high consistency) and across many different contexts (low distinctiveness), there is an increased tendency to adapt one’s initial expectations to the new outcome (accommodation). If an expectation violation happens once, but not repeatedly over time (low consistency), there is an increased tendency to treat it as an exception from the rule (immunization), especially if the expectation violation has also never happened in other contexts (high distinctiveness) and only to that particular person (low consensus).

As our results suggests, most of the coping choices are especially driven by either the main effect of low versus high consistency or the combination of (high) consistency and (high or low) distinctiveness (support for hypothesis 1b, 2b, 3b). Although we found a limited number of consensus effects on coping with expectation violations, the choice of the coping strategy was mainly predicted by distinctiveness and consistency. Thus, our results indicate that consensus might play only a minor role within the Covariation Principle, as already discussed by Kassin (1979) and Kelley and Michela (1980), at least with regard to selecting strategies for coping with expectation violations.

On a single vignette level, not every presented situation led to a significant preference for the expected strategy compared to both other strategies as well as the control group. Furthermore, there was one vignette with a significant preference for a different strategy than we expected. In this particular vignette (low consensus, low distinctiveness, low consistency), we did not find the expected preference for immunization, but a significant preference for assimilation, even after controlling for dispositional tendencies. As the preferred coping strategy did not vary between experimental and control condition, the preference for assimilation was not a response to the experimental combination of situational cues. Thus, other characteristics of the presented story seemed to promote assimilation. The specific vignette about missing a bus refers to a situation that can easily be avoided by going to the bus stop earlier. Therefore, high control over the future fulfillment of the expectation probably led to a strong preference for assimilation, as Pinquart et al. (2021) already proposed.

In general, the results of the present study support our hypotheses on an aggregated level of cue combinations, with significant preferences for the expected coping strategies against all other coping strategies, against all other cue combinations, and against the control group scores.

### 4.2. Limitations and conclusions

Of course, the results of our experimental study cannot be generalized to situations in everyday life that do not provide full information about consensus, distinctiveness and consistency. For example, in settings with a limited number of situational cues, people might use the most salient features instead (Kelley and Michela, 1980; Nisbett and Ross, 1980; Hogg and Vaughan, 2011). Even a single cue can already influence the decision about the coping strategy to a certain degree, as mentioned above (e.g., in the case of low consistency). In addition, our aim was not to find all relevant cues, but some very basic ones that can be found in many everyday situations. Further cues have been discussed in a review by Pinquart et al. (2021), such as controllability of the situation, which means that the cues from the Covariation Principle (Kelley, 1973) are certainly not the only relevant ones. Furthermore, the influence of the specific cues and attributions might be different in the case of positive expectation violations. For example, it might seem implausible to use assimilation in response to a better than expected outcome, as this would mean actively worsening the future outcome. While there might be some situations, where even this case would be plausible, future research will have to test the role of Kelley’s situational cues for coping with positive expectation violations.

As our connection of the situational cues and the ViolEx strategies seems to be suitable, one might argue whether there is also a direct connection between the coping strategies and the attribution styles included in the Covariation Principle (Kelley, 1973). According to Kelley (1973), one of three attribution styles can be observed in a particular situation, depending on the respective presence of situational cues: Internal attribution (person-related; high consistency, low distinctiveness, low consensus), external attribution (entity-related; high consistency, high distinctiveness, high consensus), or attribution to circumstances (related to the particular event and point in time; low consistency, high or low distinctiveness, high or low consensus). As a consequence of our results about cue combinations, we would expect a connection between internal attribution and accommodation, between external attribution and assimilation, and between attribution to circumstances and immunization, respectively. This might be a topic for future research. Furthermore, future studies should explore possible differences between first-person and third-person vignettes, as our study design was limited to only the latter kind of situations.

As we found a general preference for assimilation across the vignettes (in addition to the situational response shifts in coping strategies), future research should also examine whether there are individual differences in dispositional ways of coping with expectation violations, as well as possible correlates or causes of such differences. Regarding the observed general (absolute) preference for assimilation across vignettes, one might argue that this could be related to the participants’ mean age (below 30 years): As Brandtstädter and Greve (1994) suggest, younger individuals tend to use more assimilative strategies, while older people tend to change into a more accommodative mode. Therefore, with our younger sample, it is reasonable to find a certain preference for assimilation, as it is an active way to fulfill certain expectations in the future. As a consequence, future studies should examine whether there are different preferences for coping with expectation violations (e.g., toward accommodation) among older samples.

While the advantage of vignette studies as a research method is their high internal validity (because of high controllability), it is also important to reflect on their external validity, i.e., “whether the cause-effect relationship holds over variation in persons, settings, treatment variables, and measurement variables” (Shadish et al., 2002, p. 38; Eifler and Petzold, 2019). Even though we attempted to cover a broad collection of fictional settings and involved persons within our vignettes in order to optimize the generalizability, one may ask whether the vignette method leads to similar results in comparison to real situations or other response formats (such as self-reports of one’s own behavior). Eifler and Petzold (2019) found effects of social desirability in a single-vignette response format compared to the reactions from a field experiment in a propensity-score-matched analysis, featuring a frustration-reaction-scenario with a clear socially desirable outcome (i.e., not using the horn when a car keeps waiting at an already green traffic light). However, in a study by Hainmueller et al. (2015), reproducing immigrant referendum processes with different experiments, data from a multi-vignette-approach show similar results as the actual referendum data from Switzerland. Regarding expectation research, Bicchieri et al. (2021, p. 9) find “very similar” relations between expectations on social distancing compliance during the Corona pandemic and ratings of actual compliance (i.e., coping with such regulations) for both self-reports and third-person-vignettes—however, without any behavioral data as a comparison. Therefore, we conclude that, although vignette responses tend to be sometimes biased toward socially desirable answers, ratings of other people’s coping behavior should lead to similar results as self-reported coping behavior. Furthermore, the three coping options within our study do not include specific situational triggers for socially desirable responses, as we used the same general (i.e., unspecific) item phrases for each of the 16 vignettes (see methods section). The significant differences between experimental and control group also indicate that participants did not just choose a socially desirable response for each context, but rather reacted to the presence of the three cues.

Finally, it has to be mentioned that it is not clear whether the coping mechanisms might be a somewhat conscious “choice” or rather driven by automatic processes: Kelley shared the idea that people tend to be “naive psychologist[s]” (Kelley, 1973, p. 109), i.e., that they try to actively investigate the cause of events by using “a naive version of the method used in science” (Kelley, 1973, p. 109) and are aware of interdependencies between certain factors. However, he has later been criticized for his point of view, as it does not sufficiently distinguish between intentional and unintentional behavior (Malle, 1999). In our current study we do not have any measurements to reveal whether participants select coping strategies consciously or automatically. Therefore, we used the term “choice” (e.g., of coping strategies) for describing the outcome of a process, while we do not know the precise cognitive or neuronal mechanisms of these processes and whether they are conscious and intentional or not. In this particular field, further research will be necessary.

In sum, we can say that the way of coping with violated expectations is influenced by very basic situational cues. As our results show, the consistency of the expectation violating event with previous experiences, but also the distinctiveness of the situation in contrast to other contexts can shift the choice of a coping strategy toward a specific outcome: A response shift toward immunization occurs in situations with low consistency, whereas in situations with high consistency, the response shift will be toward assimilation in the case of high distinctiveness and toward accommodation in the case of low distinctiveness. Therefore, our study reveals significant connections between the ViolEx Model (Gollwitzer et al., 2018) and the Covariation Principle (Kelley, 1973).

## Data availability statement

The datasets presented in this study can be found in online repositories. The relevant files themselves can be found at OSF by using the linked website: Open Science Framework (OSF): osf.io/x4hgs.

## Ethics statement

The studies involving human participants were reviewed and approved by the Ethik-Kommission des Fachbereichs Psychologie, Philipps-Universität Marburg. The patients/participants provided their written informed consent to participate in this study.

## Author contributions

MCP developed and conceptualized the theoretical background for the study, implemented study and pre-study, analyzed the data, and wrote the first draft of the manuscript. MP supervised the study implementation and data analysis, revised the manuscript and added further sections, supervised the open science practices, and organized the funding of the project. All authors contributed to the final manuscript revision, read, and approved the submitted version.

## Funding

This study was funded by the Deutsche Forschungsgemeinschaft (DFG, German Research Foundation) (grant no. 290878970-GRK 2271, project 7), and the Open Access funding provided by the Open Acess Publishing Fund of Philipps-Universität Marburg with support of the Deutsche Forschungsgemeinschaft (DFG, German Research Foundation).

## Conflict of interest

The authors declare that the research was conducted in the absence of any commercial or financial relationships that could be construed as a potential conflict of interest.

## Publisher’s note

All claims expressed in this article are solely those of the authors and do not necessarily represent those of their affiliated organizations, or those of the publisher, the editors and the reviewers. Any product that may be evaluated in this article, or claim that may be made by its manufacturer, is not guaranteed or endorsed by the publisher.
