# Supplementary material for: Predicting coping with expectation violations: combining the ViolEx Model and the Covariation Principle
Source: Front Psychol. 2023 May 23;14:1152261. doi: 10.3389/fpsyg.2023.1152261 (PMC10242109; doi:10.3389/fpsyg.2023.1152261)
Supplement: Supplementary file 1 [file Presentation_1.PDF]

Supplementary Material

(Supplements S1, S2, S3)

Supplement S1: Exemplary Vignettes (German Wording and English Translations) and explanations of the respective cue combinations

The following exemplary vignettes (B, G, K) give an overview of our implementation of the respective cue combinations. As described in the manuscript, the combination of (high/low) consistency and (high/low) distinctiveness is relevant for our hypotheses about preferred coping strategies. However, as the original Covariation Principle also featured (high/low) consensus cues, we included these three cues in all of our vignettes (except the control condition) as we did not want to miss any unexpected results. Below, each vignette is presented in the German format (directly taken from the study) and an English translation (not featured in the study) with an additional explanation of the included cues for the experimental group. In the English vignettes (experimental group), the three cues are underlined. The control group vignettes did not include any cues related to the Covariation Principle.

Vignette B:

German wording (experimental group, assimilation cues): Heute isst Philipp mit seinen Freunden wieder einmal italienisch und bestellt sich eine Pizza. Als er die letzten beiden Male in diesem Restaurant war, hat er leider das falsche Gericht bekommen. Und auch heute bekommt er Pizza Hawaii und nicht die bestellte und daher erwartete Pizza Funghi. Das ist ärgerlich, denn so etwas passiert ihm in anderen Restaurants nicht. Als er sich umschaut, merkt er, dass seine Freunde das richtige Essen bekommen haben. Seine Erwartungen haben sich nicht erfüllt.

English translation (experimental group, assimilation cues): Today, Philipp once again visits an Italian restaurant with his friends, he orders a Pizza. The last two times at this restaurant he received the wrong meal. Today he also receives a Pizza Hawaii and not the Pizza Funghi he has ordered and expected. This is frustrating because it does not happen to him in other restaurants. As he looks around, he notices that all of his friends have received the correct dishes. His expectations did not come true.

Explanation: *In vignette B we expected assimilation as the preferred coping strategy, as it includes cues for high consistency (because Philipp also received the wrong meal at his last two visits at this restaurant) and high distinctiveness (because this does not happen to him in other restaurants). The vignette also includes a cue for low consensus (because all of his friends received the correct meals).*

German wording (control group, no cues): Heute isst Philipp mit seinen Freunden wieder einmal italienisch. Der Kellner steht am Eingang des Restaurants, hält den Gästen die Tür auf und führt sie zu einem Tisch. Philipp bestellt sich eine Pizza. Vor dem Essen geht er noch schnell zur Toilette. Kurz nachdem er wieder bei seinen Freunden am Tisch sitzt, bringt der Kellner ihnen das Essen. Doch Philipp bekommt Pizza Hawaii und nicht die bestellte und daher erwartete Pizza Funghi. Seine Erwartungen haben sich nicht erfüllt.

English translation (control group, no cues): Today, Philipp once again visits an Italian restaurant with his friends. The waiter stands at the entrance of the restaurant, holds the door open for the guests and guides them to a table. Philipp orders a Pizza. Before the meal arrives, he quickly goes to the restroom. Shortly after he arrives back at the table with his friends, the waiter serves their dishes. But Philipp receives a Pizza Hawaii and not the Pizza Funghi he has ordered and expected. His expectations did not come true.

<u>Vignette G:</u>

German wording (experimental group, accommodation cues): Leon hat heute eine Verabredung mit Tom, um Fußball zu spielen. Es wäre nicht das erste Mal, dass Tom ihn bei so einem Fußballtreffen warten lässt. Und auch wenn es um Kinobesuche oder andere Situationen geht, ist Tom immer unpünktlich. Andere von Leons Freunden sind ebenfalls oft nicht pünktlich. Trotzdem erwartet Leon, dass Tom heute pünktlich vorbeikommen wird. Doch Tom erscheint nicht pünktlich. Leons Erwartungen haben sich nicht erfüllt.

English translation (experimental group, accommodation cues): Today, Leon has an appointment with Tom to play soccer. It would <u>not be the first time Tom makes him wait at such an appointment</u>. Tom is <u>always late when they go to the cinema and also in other situations</u>. Leon’s <u>other friends are also often not on time</u>. However, Leon expects that Tom will be there on time today. But Tom does not arrive on time. Leon’s expectations did not come true.

<u>Explanation:</u> *In vignette G we expected accommodation as the preferred coping strategy, as it includes cues for high consistency (because Tom is usually late when they want to play soccer) and low distinctiveness (because Tom is also late in other situations). The vignette also includes a cue for high consensus (because other friends are also often not on time).*

German wording (control group, no cues): Leon hat heute eine Verabredung mit Tom, um Fußball zu spielen. Die beiden unternehmen oft etwas zusammen. Letzte Woche waren sie zusammen im Kino und haben sich einen Science-Fiction-Film angesehen. Heute also zieht Leon seine Fußballsachen an und holt den Fußball aus der Garage. Er erwartet, dass Tom pünktlich zum Fußballspielen vorbeikommen wird. Doch Tom erscheint nicht pünktlich. Leons Erwartung, dass Tom pünktlich erscheint, hat sich nicht erfüllt.

English translation (control group, no cues): Today, Leon has an appointment with Tom to play soccer. They often spend time together. Last week they were at the cinema and watched a science-fiction movie. Today, Leon puts on his soccer clothes and takes the football out of the garage. He expects that Tom will be there on time to play football. But Tom does not arrive in time. Leon’s expectation, that Tom would be punctual, did not come true.

Vignette K:

German wording (experimental group, immunization cues): In Stefanies WG wechseln sich alle mit den verschiedenen Putzdiensten und Aufgaben ab. Heute ist Ben dran, das Bad zu putzen. Als Stefanie zur Uni weggeht, erwartet sie, dass Ben das Bad geputzt haben wird, wenn sie zurückkommt. Die anderen in der WG erledigen ihre Arbeit immer zuverlässig, wenn sie Putzdienst im Bad haben. Beim Mülldienst und beim Kochen war auch Ben zuletzt zuverlässig. In der Vergangenheit hat er auch das Bad immer sauber gemacht, wenn er dran war. Als Stefanie diesmal jedoch nach Hause kommt, ist das Bad noch schmutzig, Ben hat noch nicht geputzt. Ihre Erwartung, dass Ben das Bad vor ihrer Rückkehr putzen würde, hat sich nicht erfüllt.

English translation (experimental group, immunization cues): In Stefanie's shared apartment, everyone takes turns at the different cleaning tasks and other duties. Today, it is Ben's turn to clean the bathroom. When Stefanie leaves to go to her student course, she expects that Ben will have cleaned the bathroom when she returns. The other roommates always reliably complete their task, when they have to clean the bathroom. When it comes to taking out the garbage or cooking, Ben was also reliable the last few times. In the past, he also always cleaned the bathroom, when it was his turn. However, when Stefanie returns this time, the bathroom is still dirty. Ben has not cleaned it yet. Her expectation that Ben would have cleaned the bathroom before her return did not come true.

Explanation: *In vignette K we expected immunization as the preferred coping strategy, as it includes cues for low consistency (because Ben always cleaned the bathroom in the past) and high distinctiveness (because Ben reliably completes other tasks like taking out the garbage or cooking). The vignette also includes a cue for low consensus (because the other roommates are reliable at cleaning the bathroom). For immunization, low consistency is the most important cue regarding the coping preference, as distinctiveness can be high or low without altering the preference for immunization (according to our hypotheses).*

German wording (control group, no cues): In Stefanies WG wechseln sich alle mit den verschiedenen Putzdiensten und Aufgaben ab. Insgesamt leben vier Personen in ihrer WG, zum Beispiel auch ihre beste Freundin Johanna, die Kunstgeschichte studiert. Die beiden kennen sich schon seit der Schulzeit und können miteinander über alles reden. Für Stefanie ist es schön, mit ihr in einer WG zu wohnen. Heute ist Ben dran, das Bad zu putzen. Als Stefanie zur Uni weggeht, erwartet sie, dass Ben das Bad geputzt haben wird, wenn sie zurückkommt. Als Stefanie später jedoch nach Hause kommt, ist das Bad noch schmutzig, Ben hat noch nicht geputzt. Ihre Erwartung, dass Ben das Bad vor ihrer Rückkehr putzen würde, hat sich nicht erfüllt.

English translation (control group, no cues): In Stefanie's shared apartment, everyone takes turns at the different cleaning tasks and other duties. Altogether, four people share the apartment, for example her best friend Johanna, who studies art history. They know each other since they went to school together and they can talk about everything. Stefanie enjoys sharing the apartment with her. Today, it is Ben's turn to clean the bathroom. When Stefanie leaves to go to her student course, she expects that Ben will have cleaned the bathroom when she returns. However, when Stefanie returns, the bathroom is still dirty. Ben has not cleaned it yet. Her expectation that Ben would have cleaned the bathroom before her return did not come true.

Supplement S2: Analysis of Manipulation Check Ratings

As the main manuscript featured only the results of the *t*-tests for the manipulation check ratings within the experimental group, but not the mean ratings themselves, those results can be found in Table S2.1:

Table S2.1. Comparison of manipulation check ratings between high and low cues

	Mean Ratings			
	High-Cue Vignettes[a]	Low-Cue Vignettes[a]	*t*	*d*
Consensus	3.39	2.21	10.65**	1.35
Distinctiveness	2.95	2.30	8.61**	1.09
Consistency	3.48	1.61	18.75**	2.38

Note. [a] Mean ratings derived from experimental group vignettes with pre-specified high versus low consensus, distinctiveness and consistency cues, respectively, *p* values resemble the results of two-tailed dependent samples *t*-tests, *d* Effect size for the comparison of the expected coping strategy rating between high and low cue ratings within the experimental group (dependent samples *t*-test, two-tailed), ** *t*-test significant at $p < .001$.

Supplement S3: Exploratory Analysis of Consensus Cues

As we had also included the consensus cues in our vignettes, though we did not have any hypotheses about possible effects, we calculated additional three-way analyses in order to reveal any unexpected effects. These results, however, have to be treated as an exploratory analysis. We therefore focus on the effects of consensus itself. For our analysis, we calculated coping strategy rating means from the respective two vignettes with the same combination of consensus, distinctiveness and consistency cues (high vs. low), independently for assimilation, accommodation, and immunization. As for our main analysis, we generally used $\alpha = .01$ as our critical cut-off for *p*-values. Additionally, within the ANOVAs and other multi-variable-tests themselves, the alpha levels were mostly adjusted by the software, using the Bonferroni method, e.g. to correct the post-hoc tests.

As a first step for a general overview of effects, we calculated three three-factor repeated-measures ANOVAs with consensus, distinctiveness and consistency (each high vs. low) as independent variables, one ANOVA per coping strategy, within the experimental group. Within these analyses, we found a significant main effect of consensus only for assimilation ratings ($F(1,61) = 13.17$, $p = .001$, $\eta_p^2 = .18$), with assimilation scores being higher in the case of low consensus ($m = .08$) than in the case of high consensus ($m = -.08$). In addition, the interaction of consensus and distinctiveness was significant for assimilation ($F(1,61) = 7.79$, $p = .007$, $\eta_p^2 = .11$), with the highest assimilation scores for the combination of low consensus and low distinctiveness ($m = .14$), and immunization ($F(1,61) = 14.12$, $p < .001$, $\eta_p^2 = .19$), with the highest immunization scores for the combination of low consensus and high distinctiveness ($m = .30$). Furthermore, a significant three-factor interaction of all three cues was found for immunization ($F(1,61) = 37.63$, $p < .001$, $\eta_p^2 = .38$), with the highest immunization scores for the combination of low consensus, high distinctiveness and low consistency ($m = 1.31$); any other possible effects of consensus remained non-significant. However, all of these ANOVA results do not directly affect our hypotheses about the actually preferred coping strategies (i.e. the highest ratings for each cue combination), as the effects also include (and might be driven by) response shifts within the less preferred coping strategies. From a practical perspective, one can assume that mainly the most-preferred coping strategy would be behaviorally relevant, while the less preferred would be less likely to be observed, especially in the case of any significant differences between strategy preferences. Therefore, we continued our analysis with a focus on the direct comparison of ratings within and between the respective cue combinations.

As our next step, we tested whether there were differences in the preferred coping strategies between the high and low consensus variants of the respective “distinctiveness x consistency” cue combinations. Taking a closer look at these cue combinations themselves within further one-way ANOVAs, we found that there was no significant difference for assimilation ratings between the variants of “distinctiveness high, consistency high” with high vs. low consensus, as we had expected. Both ratings were significantly higher than almost every other cue combination ($F(7,427) = 46.35$, $p < .001$, $\eta_p^2 = .43$), apart from non-significant differences with the cue combination “consensus low, distinctiveness low, consistency high”. For accommodation, there was no significant difference between the variants of “distinctiveness low, consistency high” with high vs. low consensus, as we had expected. These two ratings were also significantly higher than the accommodation ratings in every other cue combination ($F(7,427) = 24.91$, $p < .001$, $\eta_p^2 = .29$). For immunization, all ratings with “low consistency” cues were significantly higher than ratings with “high consistency” cues, as expected ($F(7,427) = 141.709$, $p < .001$, $\eta_p^2 = .70$). However, there were some significant differences within the cue combinations containing “low consistency”: The significantly highest values were found for “consensus low, distinctiveness high, consistency low”, whereas the differences between the other cue combinations

where only partially significant in some comparisons: Ratings for “consensus high, distinctiveness high, consistency low” were significantly higher than “consensus low, distinctiveness low, consistency low”, whereas “consensus high, distinctiveness low, consistency low” was in between both, but not significantly different. The results are shown in Figure S3.1.

As a last step, we also analyzed the differences between the expected coping strategies and other possible strategy ratings within each cue combination. An overview of these results can be found in Table S3.1. For seven of eight cue combinations, the expected strategy was significantly higher than both other strategies, whereas for “consensus low, distinctiveness low, consistency high” the expected strategy (accommodation) received a higher score than only one of the two other strategies (immunization, but not assimilation). Comparing the expected coping strategies in the experimental condition with the control condition, we found significant differences in the expected direction for six of the eight cue combinations, whereas there were no significant differences for “consensus high, distinctiveness high, consistency high” (assimilation) and for (consensus low, distinctiveness low, consistency low) (immunization). The detailed results can be found in Table S3.1.

Figure S3.1. Strategy ratings depending on three-cue combinations

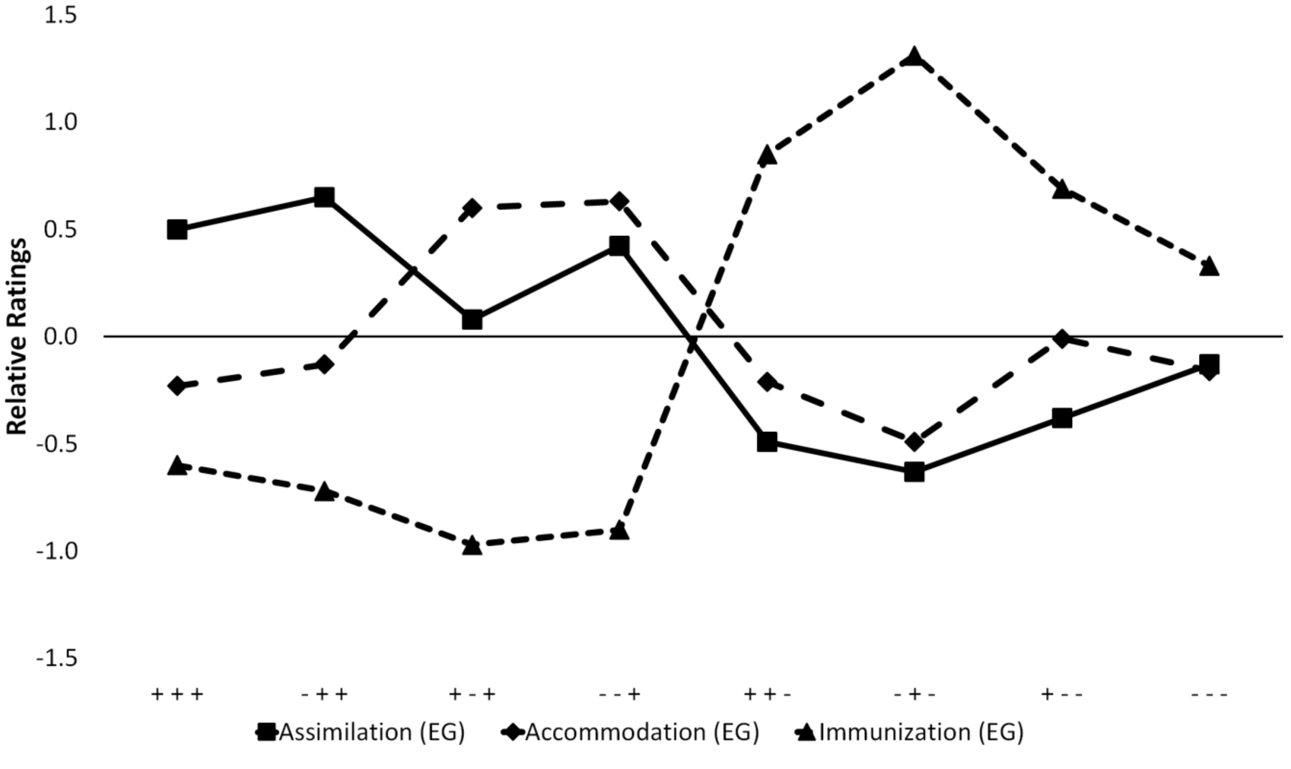

Note. The figure shows the relative ratings for each coping strategy in the experimental group for the possible combinations of consensus, distinctiveness, and consistency, respectively, each high or low. The y-axis shows the deviation from each mean rating, a value of zero represents the mean rating across all eight cue combinations.

Table S3.1. Analysis of ratings within three-factor cue combinations

Cues[a]	Expected coping strategy	Ratings in experimental group (One-way ANOVA)				Control group (CG) comparison[b]	
		Ass.	Acc.	Imm.	η_p^2	Rating of expected strategy in CG	*d*
+ + +	Assimilation	*.50*[cd]	-.23[c]	-.60[d]	.45	.29	.41
- + +	Assimilation	*.65*[cd]	-.13[c]	-.72[d]	.59	.16**	.90
+ - +	Accommodation	.08[c]	*.60*[cd]	-.97[d]	.67	.23**	.68
- - +	Accommodation	.42	*.63*[d]	-.90[d]	.66	.29**	.59
+ + -	Immunization	-.49[d]	-.21[c]	*.85*[cd]	.54	-.32**	1.78
- + -	Immunization	-.63[d]	-.49[c]	*1.31*[cd]	.75	.19**	2.12
+ - -	Immunization	-.38[d]	-.01[c]	*.69*[cd]	.43	.06**	1.04
- - -	Immunization	-.13[c]	-.16[d]	*.33*[cd]	.20	.26	.13

Note. The means of expected coping strategies within the experimental group are formatted in italics. [a] Pre-specified combination of consensus, distinctiveness, and consistency, respectively, each high or low, [b] Rating of the hypothesized coping strategy in the control group. The asterisks indicate whether the this rating differs significantly between experimental and control group (independent samples *t*-Test, one-tailed), [c] Significant difference between highest and second highest rating at $p < .01$, [d] Significant difference between highest and lowest rating at $p < .01$, η_p^2 Effect size for the comparison of ratings within the experimental group (one-way ANOVA), *d* Effect size for the comparison of the expected coping strategy rating between experimental group and control group (independent samples *t*-test, one-tailed), ** *t*-test significant at p < .001.
